# Implementation fidelity, attitudes, and influence: a novel approach to classifying implementer behavior

**DOI:** 10.1186/s43058-022-00307-0

**Published:** 2022-06-06

**Authors:** Taren Swindle, Julie M. Rutledge, Janna Martin, Geoffrey M. Curran

**Affiliations:** 1 Department of Family and Preventive Medicine, University of Arkansas for Medical Sciences, 4301 W. Markham St., #530, Little Rock, AR 72205-7199 USA; 2 Education and Research in Children’s Health Center, College of Applied and Natural Sciences, Louisiana Tech University, Ruston, USA; 3 Department of Pharmacy Practice and Psychiatry, University of Arkansas for Medical Sciences, 4301 W. Markham St, Little Rock, AR #522-472205-7199 USA; 4 Central Arkansas Veterans Healthcare System, 4300 W 7th St, Little Rock, AR 72205 USA

**Keywords:** Implementation, Adoption, Behavior, Classification, Childcare, Obesity prevention, Implementation science, Early care and education, Nutrition

## Abstract

**Background:**

The current study sought to (1) describe a new classification approach for types of implementer behavior and (2) explore the implementer behavior change in response to tailored implementation facilitation based on the classifications.

**Methods:**

A small-scale, cluster-randomized hybrid type III implementation trial was conducted in 38 early care and education classrooms that were part of the Together, We Inspire Smart Eating (WISE) program. WISE focuses on 4 evidence-based practices (EBPs), which are implemented by teachers to promote nutrition. External facilitators (*N* = 3) used a modified Rapid Assessment Procedure Informed Clinical Ethnography (RAPICE) to complete immersion (i.e., observations) and thematic content analyses of interviews to identify the characteristics of teachers’ behavior at varying levels of implementation fidelity. Three key factors—attitudes toward the innovation, fidelity/adaptations, and influence—were identified that the research team used to classify teachers’ implementation behavior. This process resulted in a novel classification approach. To assess the reliability of applying the classification approach, we assessed the percent agreement between the facilitators. Based on the teachers’ classification, the research team developed a tailored facilitation response. To explore behavior change related to the tailored facilitation, change in fidelity and classification across the school year were evaluated.

**Results:**

The classifications include (1) enthusiastic adopters (positive attitude, meeting fidelity targets, active influence), (2) over-adapting adopters (positive attitude, not meeting fidelity targets, active influence), (3) passive non-adopters (negative attitude, not meeting fidelity targets, passive influence), and (4) active non-adopters (negative attitudes, not meeting fidelity targets, active influence). The average percent agreement among the three facilitators for classification was 75%. Qualitative data support distinct patterns of perceptions across the classifications. A positive shift in classification was observed for 67% of cases between the mid-point and final classification. Finally, we generated an expanded classification approach to consider additional combinations of the three factors beyond those observed in this study.

**Conclusions:**

Data from this study support the ability to apply the classification approach with moderate to high reliability and to use the approach to tailor facilitation toward improved implementation. Findings suggest the potential of our approach for wider application and potential to improve tailoring of implementation strategies such as facilitation.

## Contributions to the literature

- This study provides a novel classification approach for implementer behavior that can inform tailoring of facilitation and other implementation strategies. Specifically, our team developed a tailored facilitation response to address the influence, attitudes, and skills of the implementers reflective of their classification.
- Our data illustrates that not all low fidelity scores are equal; differences in implementer attitude and adaptations can help to understand low fidelity.
- Non-adopters in our study faced greater perceived contextual barriers, underscoring prior literature on the strong influence of context on implementation.

## Background

Diffusions of innovation (DOI) [1], a central theory of implementation science, classifies implementers as fitting into 5 categories relative to the rate of diffusion: (1) innovators, (2) early adopters, (3) early majority, (4) late majority, and (5) laggards. Innovators are the smallest category at an estimated 2.5% of adopters, have the quickest rate of implementation uptake, and are described as venturesome. Early adopters follow closely behind innovators in implementation and are characterized by respect in their social networks and a reputation for making judicious, deliberate choices. Late adopters, the 4th group to implement, are known for being skeptical of change. Finally, laggards are traditional individuals with the slowest rate of implementation, characterized by a resistance to change and, frequently, a need for pressure to adopt new innovations. Socioeconomic status, personality characteristics, and communication behaviors predict an implementer’s placement in these categories with those of higher socioeconomic status, certain personality traits (e.g., empathy, intelligence, rationality, risk-tolerant) and greater social participation demonstrating earlier adoption [2]. The value of the theory of diffusions of innovation (DOI) has been demonstrated across hundreds of studies over five decades [3], suggesting a classification of implementers has utility for informing implementation efforts. Specifically, targeting implementation strategies to known classifications of implementers holds promise as an approach for tailoring implementation strategies.

Additional characteristics of implementer behavior beyond the speed of implementation may improve the classification of adopter behavior. Specifically, DOI addresses the speed of adoption but not the quality of adoption. Pairing the DOI with other implementer characteristics may be one promising pathway to more nuanced understanding of implementation. A conceptual framework for implementation by Carroll and colleagues [4] proposes that interventions lead to desired outcomes through adherence to the content, frequency, and duration of the intervention as designed (i.e., fidelity). The framework also details potential moderators that may affect the relationship between intervention exposure and adherence (e.g., policy descriptions, implementation strategies, and participant responsiveness) [5]. This suggests that how an implementer thinks about and uses an EBP may be influential for ultimate outcomes. Integrating these facets with DOI to provide an improved classification of implementer behavior has the potential to advance tailoring of implementation strategies and, ultimately, desired implementation outcomes (e.g., adoption, fidelity, sustainment).

Based on the combination of the DOI and the conceptual framework for implementation fidelity, the attitudes, adaptations, and influence of implementers are potentially salient factors that could improve the classification of implementer behavior. Implementers’ attitudes (i.e., participant responsiveness) toward the targeted evidence-based practices (EBP) likely affect uptake and quality of delivery [6]. For example, teachers’ perceptions of the appeal of an EBP have demonstrated significant relationships with their use of EBPs [7]. Similarly, attitudes towards a healthcare initiative (i.e., wait time reduction) have predicted participation in the change effort among staff in primary care clinics [8]. Another potential factor is the type of adaptations made by implementers (i.e., deviations from prescribed content, frequency, and duration). Some implementers make fidelity-consistent adaptions to improve the fit of an innovation within a context; other implementers make fidelity-inconsistent adaptations that change or remove core components of the intervention in a way that is likely to reduce effectiveness [3]. Furthermore, research suggests attitudes about EBPs may be related to the types of adaptations implementers make [9, 10]. In the mental health setting, therapists with negative attitudes toward EBPs have been shown to make more adaptations of reducing and rearranging (i.e., not using some components or using them in a different sequence) [9] while openness to EBPs has resulted in more adaptations of augmentation (i.e., adding to the innovation) [10]. As recognized by DOI, social networks are key for influencing the spread of innovations. Key opinion leaders’ promotion of EBPs has been linked with improved uptake of mental health practice of teachers [11], use of HIV self-testing and treatment [5], and use of clinical guidelines by health professionals [5]. Conversely, opinion leaders can be hostile or ambivalent towards an EBP [12, 13]; although this effect has been studied to a lesser extent. Thus, the attitudes and adaptations an implementer make can have a reaching impact on system implementation based on their influence in the context. Understanding the attitudes, adaptations, and influence of implementers may provide valuable information beyond consideration of whether uptake occurs and the speed of uptake.

The aim of this study is twofold. First, we present data from a mixed methods study to illustrate a novel classification approach for implementer types. This classification approach incorporates multiple implementer characteristics (attitude, fidelity/adaptations, and influence) and suggests actionable grouping of responders based on these factors. We illustrate the use of this classification approach to guide implementation facilitation efforts to leverage the strengths of each recipient and to overcome barriers to implementation across multiple theoretical domains (e.g., personal skills, inner/outer context) and implementation determinants (e.g., organizational leadership) while building capacity and creating buy-in. Second, we examine the changes in implementer behavior in response to tailored implementation support based on the classification approach to assess its value for informing implementation efforts. Our work provides an example of real-world consideration of “multiple inputs” on behavior [14] and tailoring of facilitation in light of these inputs to promote behavior change. In so doing, this work suggests a tangible approach for demystifying the “black box” [15] of facilitation.

## Methods

### Design

A two-arm, small-scale, cluster-randomized hybrid type III implementation trial was conducted in 38 early care and education classrooms to test “enhanced” vs. “basic” stakeholder-selected strategies to implement evidence-based practices (EBPs) of a healthy eating intervention in early care and education (ECE) classrooms. Formative research included input from key stakeholders on determining key barriers and facilitators, prioritizing the most important and feasible implementation strategies, and operationalizing the multi-faceted implementation strategy package for delivery in the enhanced condition [16, 17]. The full description of the intervention components, implementation strategies, timeline of activities, and results of the trial are published elsewhere [17]. As reported prior, the total turnover rate among teachers in the sample was 43%. [17].

### Participants

The study took place in the USA in a southern state at an urban head start in the 2018–2019 school year. ECE teachers in the enhanced implementation condition are the focus of this study (4 sites, 20 classrooms, 35 teachers); these teachers received a package of multi-faceted implementation strategies as detailed below. All study activities were approved by the IRB at the University of Arkansas for Medical Sciences.

### Intervention

Together, We Inspire Smart Eating (WISE) is a nutrition curriculum designed to promote acceptance and intake of 8 target fruits and/or vegetables (F/V) [18]. WISE is comprised of 4 EBPs: (1) hands-on food experiences in small groups, (2) use of an owl mascot puppet, (3) teacher role-modeling, and (4) positive feeding practices. ECE teachers are the implementers of the WISE intervention in their classrooms. All materials and training to implement the program were provided as well as resources to support parent engagement. Materials include a manual with 6 + lesson options and classroom activities for each of the 8 WISE units, the WISE mascot puppet, basic cooking tools (e.g., measuring cups, child-safe knives), and high-quality color copies of parent resources. Research on WISE demonstrates increased F/V consumption in 3- to 5-year-olds and decreased intake of junk foods (e.g., chips, cakes, cookies, candies) [19, 20].

### Enhanced support: implementation strategy package

The multi-faceted, enhanced support package of strategies is detailed in our prior work; stakeholders had input into the selection and specification of all strategies [17] (Table 2). The package included strategies aimed to support the context (trained internal champions; formal commitment with leadership; blueprint to guide implementation) and ECE teachers as implementers (reminder of WISE EBPs on cutting boards, incentive program for meeting fidelity markers, tailored educational materials based on EBP fidelity). Site champions were classroom teachers who volunteered or were asked by their site leader to serve as the local champion for WISE and received extra training to help support WISE and advocate for the program among their peers. Observational data from mealtime and food experiences were analyzed on a quarterly basis to inform the delivery of implementation strategies. For example, incentives were given to those reaching fidelity in each EBP per quarter. Furthermore, educational resources started with handouts to reinforce EBPs then moved to educational videos to further solidify the concepts.

In addition, facilitation was a “meta-strategy” supporting both the teachers through direct coaching and the context through assessment and trouble-shooting. Facilitation is “a process of interactive problem solving and support that occurs in a context of a recognized need for improvement and a supportive interpersonal relationship” [21]. The facilitators had resources (i.e., the facilitator toolkit) ready to use as needed to reinforce the 4 WISE EBPs including evidence pitches, testimonials, handouts, and videos. Facilitator 1 was a prior classroom teacher with over 20 years of classroom experience; facilitator 2 was also a prior teacher in the early care and education setting as well as the PI of the study with expertise in implementation science; facilitator 3 had experience as a community advocate and coaching children. Facilitators 2 and 3 completed the Veteran’s Affairs Quality Enhancement Research Initiative training in implementation facilitation, adapted it for use with WISE, and shared the content with facilitator 1. The PI supported bi-weekly reflection on the facilitation process as an opportunity for ongoing training and growing in consistency of approach between facilitators.

### Data collection

#### Fidelity

After demonstrating 85% reliability with gold standard observers on both video and live classroom observations, data collectors assessed WISE intervention delivery in the fall, winter, and spring of the school year using the WISE Fidelity [22] tool. The tool captures the details of the content of the intervention delivered and the duration of lessons [5]. Specifically, the WISE fidelity measure has multiple items designed to measure the delivery of each of the EBPs on a 1 to 4 scale (1 = not at all, 4 = very much). For each point on the scale, data collectors are trained to assess for discrete behaviors to support their score (e.g., 1 = teachers did not try the food with the children, 4 = teacher tried the food with all groups). An average score of 3 for items related to each EBP is conceptualized as achieving fidelity. ECE teachers achieving this level of fidelity were incentivized; ECE teachers below this level received facilitation and tailored educational resources to increase fidelity for each EBP as needed. Fidelity-consistent adaptations include additions, deletions, and or revisions to the program that retain the core components as designed. For WISE, documented examples of fidelity-consistent adaptations include adding costumes or special voices for the WISE mascot and having small groups do their lessons on different days of the week. Fidelity-inconsistent adaptions are those that are incompatible with delivery of the EBPs for the desired impact. WISE examples of fidelity-inconsistent adaptations include combining classrooms to deliver the lesson, using the mascot to pressure children, and adding extra salt and sugar to recipes.

#### Formative qualitative interviews

At the end of the fall and winter quarter, ECE teachers in the enhanced condition showing the lowest fidelity in the four components were identified for interviews (*N* fall = 15; *N* winter = 8). These teachers were behind fidelity targets in 3 or more of the WISE practices. Positive feeding practices were the WISE component with the most teachers struggling to achieve fidelity while role modeling was the practice with the highest overall fidelity [17]. At each time point, 5 teachers from this pool were randomly selected for interviews for a total of 10 formative interviews led by a WISE facilitator. The interview guide was informed by constructs from the Integrated Promoting Action on Research Implementation in Health Services (i-PARIHS) framework and designed to elicit barriers to implementation fidelity (see Table 1). The facilitator who completed the interview also completed a lesson observation with each interview participant and reviewed fidelity data from self-report measures. Specifics of these measures can be found elsewhere [22]. In addition to the 10 interviews with low-performing teachers, qualitative interviews included site leaders (i.e., those providing local oversight) and site champions who had not already been interviewed at the end of the intervention year (*N* = 5).

**Table 1:** Example interview questions by iPARIHS construct

iPARISH construct	Sample questions
Recipient experiences	• Tell me the best/worst thing about your experience with WISE so far. • How do you prepare for a food experience?
Context	• Tell me about your director’s role with WISE. • How often do you talk with other teachers at your center about WISE? What do they have to say?
Implementation support/facilitation	• In October, WISE delivered educational handouts to support your WISE activities. *Show examples.* Specifically, your classroom received “tailor based on their delivery.” Tell me about your use of these resources. What would you change about the handouts to make them more useful or appealing to you? • What support would you like to get from WISE staff that you are not getting?
Innovation barriers	• Eating and enjoying food in front of the kids, or role modeling, is an important WISE practice. What is the hardest part about role modeling for you? What could WISE do to support you better in role modeling with children? • Windy is the mascot for the WISE curriculum. What is the hardest part about using Windy for you? What could WISE do to support you in using Windy in your classroom?

### Data analyses

#### Qualitative coding

For the purposes of this study, external facilitators used a modified Rapid Assessment Procedure Informed Clinical Ethnography (RAPICE) [23] of moderate intensity to complete immersion (i.e., observations) and thematic content analyses of interviews to understand the characteristics of teachers at varying levels of intervention fidelity. RAPICE is a pragmatic approach to collecting and using qualitative data in the context of an ongoing trial [23]. RAPICE includes prescribed steps to document site visits and study activities including field notes, informal and semi-structured interviews, member checking, triangulation, peer debriefing, and prolonged engagement. [23].

First, the PI and two staff (one junior coder, an undergraduate psychology student; one senior coder, a research assistant with 5 + years of experience with WISE) collaborated to create a codebook using interview examples. Specifically, the team deployed directed content analysis [24] in which we coded barriers and facilitators for each implementation strategy and for WISE on the whole. Team members coded interviews to identify concrete codebook examples for all codes; this continued until the team reached consensus on the meaning and application of codes (*N* = 3 interviews). The junior coder completed all coding after consensus was established; all decisions were reviewed by the senior coder, and any disagreements were decided by the PI.

Next, the PI and the senior coder, both were involved in developing and applying the classification approach, read the interviews to identify the examples of each adopter type. The coders were blind to the participants’ classification at this step. After the coding of this step was complete, the research team checked the selected quotes against the classification of the participant. Finally, the coding team examined the barriers and facilitators mentioned by each adopter type to determine if similarities and differences were present between the groups.

#### Developing classifications

As the research team performed observations and interviews designed to iterate and tailor the implementation strategies, patterns were noted in three key areas: (a) attitudes (e.g., supportive versus resisting), (b) fidelity/adaptations (e.g., high fidelity with fidelity-consistent or no adaptations, low fidelity with no adaptations or fidelity-inconsistent adaptations), and (c) influence (e.g., active versus passive). For the initial classification (after the fall interviews), the three facilitators used discussion of their experiences, recollection of specific behaviors, interview content, and consensus building to classify participants in these three domains. This process resulted in 4 groups. (1) Enthusiastic adopters exhibited supportive attitudes towards WISE and positive influence in their setting; any adaptations were fidelity-consistent, and fidelity targets were demonstrated. (2) Over-adapting adopters, while similarly exhibiting positive attitudes toward WISE, made fidelity-inconsistent adaptations [3] that were potentially detrimental (e.g., using mascot to shame children) resulting in failure to meet fidelity targets. Yet, this group was frequently vocal about their positive experience with WISE. (3) Passive non-adopters demonstrated did not meet fidelity targets and showed a lack of interest in adopting WISE or receiving facilitation support; few to no adaptations were noted. This group did not try to influence their peers in regard to WISE. (4) Active non-adopters were vocal about their complaints in adopting WISE and/or noticeably against receipt of facilitation support; most active non-adopters did not meet fidelity targets and made no adaptations. We tested our shared understanding of the characteristics of each discrete group by re-classifying teachers at the end of the school year (after another 6 months of implementation support including tailored facilitation). Mid-point classifications were completed collaboratively between the 3 facilitators and data coordinator as criteria for each category were refined.

#### Assessing reliability in classifications

At the end of the school year, the 3 facilitators provided independent ratings of adopter classifications for reliability assessment. We assessed the reliability of these classifications using percent agreement. Percent agreement is the absolute number of agreements divided by the total number of classification ratings (i.e., the percent of classifications that are in agreement) [25].

#### Fidelity change

Fidelity assessments were collected during the fall, winter, and spring quarters of the school year. We assessed the changes in the percentage of classrooms achieving fidelity between each time point to examine patterns that may be attributed, at least partially, to the shift in facilitation approach in the winter of the school year (i.e., greater improvements from winter to spring than from fall to winter would support the shift in approach).

### Tailored facilitation response

In response to the real-time needs of the project and ongoing analyses, external facilitators decided upon a two-prong approach to resuming facilitation activities after the holiday break (i.e., January of the school year and following). First, facilitators focused on active non-adopters. Specifically, external facilitators determined to build stronger relationships with active non-adopters, trying to understand rather than change their behavior with a secondary goal of reducing their vocal negativity within the group. Rather than reinforcing the WISE components through the delivery of incentives or educational materials, coaches found reason to affirm each teacher in small ways related to their professional identity (but outside the innovation). With over-adapting adopters, facilitators worked to set goals collaboratively with the teachers guiding them to focus on one specific practice and to attend to the emotion and process contributing to the fidelity-inconsistent adaptations and by using open-ended questions, active listening, reflections to elicit teachers’ ideas for change, and planning to support change. Facilitators attempted to build interest among passive non-adopters by building self-efficacy and highlighting WISE successes. For all non-adopters, shifts along the categorical spectrum became the goal rather than a narrow focus on achieving fidelity (e.g., transitioning some teachers from active non-adopter to passive non-adopter, passive non-adopter to adopting). Second, the facilitators attempted to leverage and encourage enthusiastic adopters, praising them for the successes both directly and to the supervisor and asking for their support to increase buy-in at the site. Eliciting enthusiastic adopters’ support included asking them to check in with and offer help to non-adopting teachers as well as sharing their success stories with their peers. Connections between enthusiastic adopters and passive non-adopters were prioritized to increase interest in the innovation. These strategies were iterated between the fall and spring of the school year based on collaborative discussion among facilitators about what worked in the classroom, drawing on the i-PARIHS concept of validating “real-world” evidence [26] for teachers, motivational interviewing strategies, and behavior change techniques seeming to fit with each category.

## Results

### Classifications

**Fig. 1:**
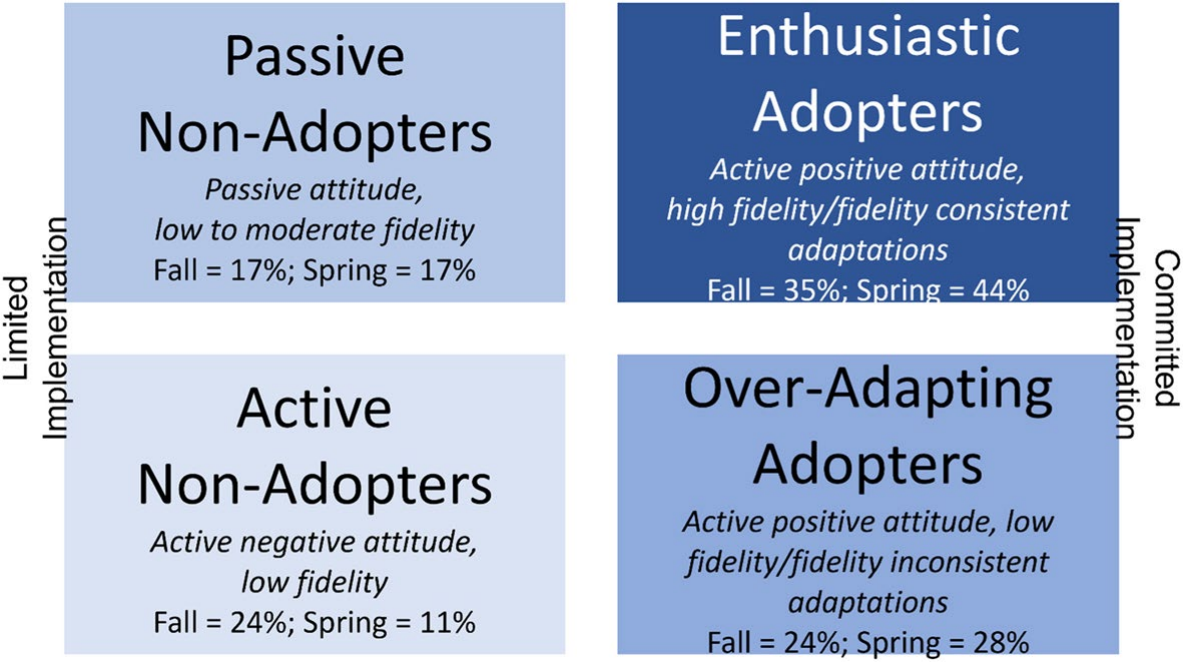
Adopter types at fall and spring assessments

Classifications at mid-point (i.e., fall) were as follows: enthusiastic adopters (35%), over-adapting adopters (24%), passive non-adopters (17%), and active non-adopters (24%) (see Fig. 1). These categorizations informed tailored facilitation for the remainder of the school year. Selecting classifications with agreement for final classification at the end of the school year (i.e., spring) yielded the following: enthusiastic adopters (44%), over-adapting (28%), passive non-adopters (17%), and active non-adopters (11%). At the final classification, facilitators 1 and 2 exhibited 67% agreement on their classifications, facilitators 2 and 3 exhibited 93% agreement on their classifications, and facilitators 1 and 3 exhibited 64% agreement. Overall, the average percent agreement was 75%.

Based on the shared classifications and consensus of facilitators 2 and 3, a positive shift in the responder category was observed for 67% of cases remaining in the sample between the mid-point and final classifications. Specifically, shifts reflect a 9% increase in enthusiastic adopters and a 7% decrease in active non-adopters. There were three cases where a shift was in an undesired direction.

### Fidelity

**Fig. 2:**
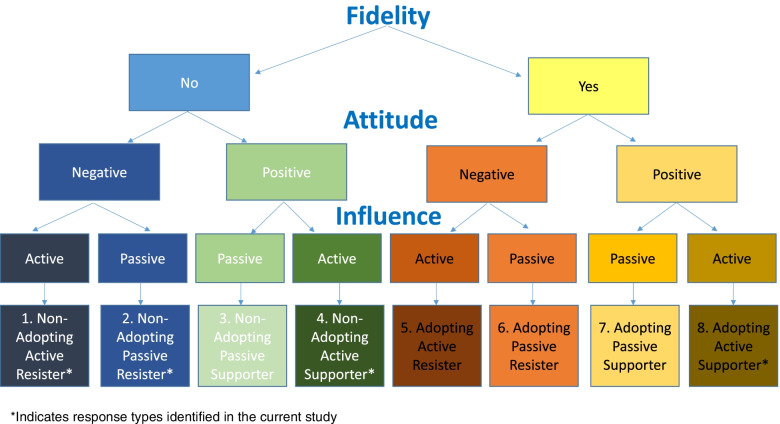
The Fidelity, Attitudes, Influence, Typologies (FAIT) classification approach

Changes in fidelityacross time have been reported prior [17] (Fig. 2). In every case, the enhanced group receiving the multi-faceted implementation strategy was higher in fidelity than the basic support group at the end of the school year. For example, fidelity to hands-on exposure decreased at every assessment for the basic group and increased at every assessment for the enhanced group for a total difference of 27% at the spring assessment. Notably, the change from time point 2 to time point 3 was largest for hands-on exposure and role modeling for the enhanced group. Given that our facilitation strategy was the most iterated aspect of the implementation strategy package over the year, these EBPs seem to be well supported by our tailored facilitation response.

### Qualitative feedback by adopter type

Reflective of the design of our study, two of the four enthusiastic adopters in the interview sample were also site champions. The remaining classifications were split between over-adapting adopters (*n* = 4, one of which was a site champion), passive non-adopters (*n* = 3), and active non-adopters (*n* = 1). Interviews with enthusiastic adopters indicated value for the intervention’s positive effect on children, responsiveness to the implementation strategy support, and individual preparation for lesson delivery. Although passive non-adopters had some positive things to say about the intervention, a majority of their comments focused on the context of the site including descriptions of the experience of being the only teacher in a classroom and having a supervisor who provided limited support. Active non-adopters described being pushed to do WISE without understanding from leadership, argued for alternatives to the EBPs of WISE, and often did not know about or understand the implementation strategy support. Over-adapting adopters were overwhelmingly positive about the intervention with comments in this respect that mirror that of enthusiastic adopters. However, over-adapting adopters frequently justified their departure from WISE EBPs in their interviews (see Table 2 for example quotes by each adopter type).

**Table 2:** Exemplar quotes by adopter types

Adopter type	WISE	WISE implementation strategies	Context
Active non-adopter	• They want us to push this WISE and we don’t have time for WISE and [TSG] … All that stuff and they just insist that we do WISE WISE WISE. It’s something come before WISE, and you get mad if we don’t have this paperwork together. But you want us to stop what we doing to do some WISE	• You have to get WISE in and you have to get this in. And then people coming in to observe you [facilitation]. It’s like the magnifying glass is on you. You know…and then they are going to find something wrong. You not doing this the right way	• They (leadership) always be coming in with all this stuff we were gonna have to do…I felt like if this is not your class. You just go on, you just come and do whatever, and it was just overwhelming to me
Passive non-adopter	• Well we don’t, well I don’t really you know ask them if you’re full or are you hungry. They will just let us know you know if they you know want more…I just go with them. I don’t really say are you full or are you still hungry or whatever. They just usually let us know	• “I did, she [facilitator] gave me some handouts. I have them up there when my WISE little thing… I have looked at some of them.”	• Being the only teacher in here…Explaining and all that kind of fell on me to do the whole thing…
Over-adapting adopter	• [For Windy] You don’t even really have to have a little voice…That’s why I don’t do it, I don’t bring it out right at the beginning. I sit here for a few minutes just to see who’s gonna start, and then I say, “well, let me see…” And then I look, just kinda be looking…. And one little boy, he don’t hardly wanna try nothing. But then when I got up and go that, and “woo, I’ll taste mine!” Yes. To make sure we gonna get everybody into it, we gonna try Windy. And Windy kinda works. Before you know it, everybody eating	• I was very pleased with how they [facilitators] would come out to the center and talk with me to see if there was anything that I needed, and went to the classrooms to check on the teachers to see how things were going, was there anything that they could bring in, I mean I just could not believe how they just tagged onto us like we was just a part of you all’s entire program!	• Probably about the hardest thing is like, one, a couple of times we didn’t get the WISE food…. when the kids get used to but we have to skip one, then we have to tell them why. So that’s about the hardest thing
Enthusiastic adopter	• “I mean it’s been, it’s been great. I mean the kids enjoy it, and the teacher’s enjoy it, and actually they learn a lot about the different size, different color…how it grows, where it comes from, from a tree, from a garden. They, it’s, I think it’s helping the kids out. They be talking about it. They go home and talk to their parents about it, which makes me happy.”	• And after we had that big meeting [champion led discussion] and I came back we tried that small group and we were like okay, yeah this is where it’s at… once we sat down and we tried the small groups it really worked out and it more calm, you know it was better and the kids were more one-on-one, and you could get them to try it	• She, (director) now she helps us plan, you know, make sure it’s planned… And she let’s us know ahead of time what we need. We know what day. And we make sure, she makes sure we got it on our lesson plan so the parents can be able to know what activity we give ‘em to work on that week

Adopter types also reported unique perceptions of strategies, WISE, and their context. Table 3 presents patterns of perceptions of the implementation strategies, innovation, and context by response type. For example, most enthusiastic adopters were positive about all strategies, WISE as a whole, and feelings about their context; only the facilitation strategy did not have a clear positive valence in the perceptions for this group. Similarly, over-adapting adopters held positive perceptions in all areas except toward their context where views were neither overly positive nor negative. Passive non-adopters had negative perceptions of the cutting board (e.g., wanted to change dimensions), facilitation (e.g., wanted more feedback), and their context (e.g., not enough teachers to support implementation). Active non-adopters held negative views of the cutting board (e.g., did not know about it), incentives (e.g., unfairly contributed to “a bad report”), facilitation (e.g., made them feel “under the microscope,”), and the context (e.g., lack of support/supplies). While the non-adopting groups saw the value of the WISE intervention, they were less positive than adopting types. Specifically, enthusiastic adopters and over-adapting adopters had over 30 unique comments about their positive perceptions of the WISE intervention, particularly its positive effect on children. Non-adopters had fewer than 10 positive comments. These patterns reflect distinct experiences across response types.

**Table 3:** Overall patterns by type

Interview topic	Active non-adopter	Passive non-adopter	Over-adapting adopter	Enthusiastic adopter
*EBP reminder cutting board*	−	−	+	+
*Champion*	o	+	+	+
*Handouts*	o	o	+	+
*Videos*	o	+	+	+
*Incentives*	o	+	+	+
*Coaching*	−	−	−	o
*WISE innovation*	+	+	+	+
*Context*	−	−	−	+

Commitment form and blueprint were for site leadership and did not apply to teachers

“ + ” = opinions were positive; “ − ” = opinions were negative, “o” = opinions did not follow a clear pattern

### Expanded classification approach

After the conclusion of the study, the research team considered the potential for additional combinations on the factors of fidelity/adaptations, attitude, and influence. All logical combinations of these factors were considered beyond the 4 key ones noted in our fieldwork in ECE. Figure 2 reflects our conception of all possible combinations of these factors, the Fidelity, Attitudes, Influence Typologies (FAIT). In total, we conceive 8 possible classifications. Fidelity is the driving factor in our approach; fidelity is observable and will reach targeted thresholds or not (i.e., adopting, or non-adopting). Next, attitude towards EBPs is classified as positive or negative; this categorization could reflect validated measures [27] and/or field interactions. Finally, we conceptualize that influence will be active or passive; sociometric nominations [28], self-assessments [29], and/or field interactions/direct observations could be used to determine an implementer’s influence in a given context. Of the 8 possible combinations, we have designated the 4 we observed in our study, which have been renamed in Fig. 2 to reflect the naming conventions of the full classification system rather than the labels the study team used during the ongoing study. Specifically, the “enthusiastic adopters” type is equivalent to “adopting active supporters” in the revised expanded classification approach, “active non-adopter” is equivalent to “non-adopting active resister,” “passive non-adopter” is equivalent to “non-adopting passive resister,” and “over-adapting adopter” is equivalent to “non-adopting active supporter.” This expanded approach illustrates how an implementer can be adopting or non-adopting (i.e., not meeting fidelity targets), have an active or passive influence in the context, and be supporting or resisting in their attitudes towards the intervention.

**Fig. 3:**
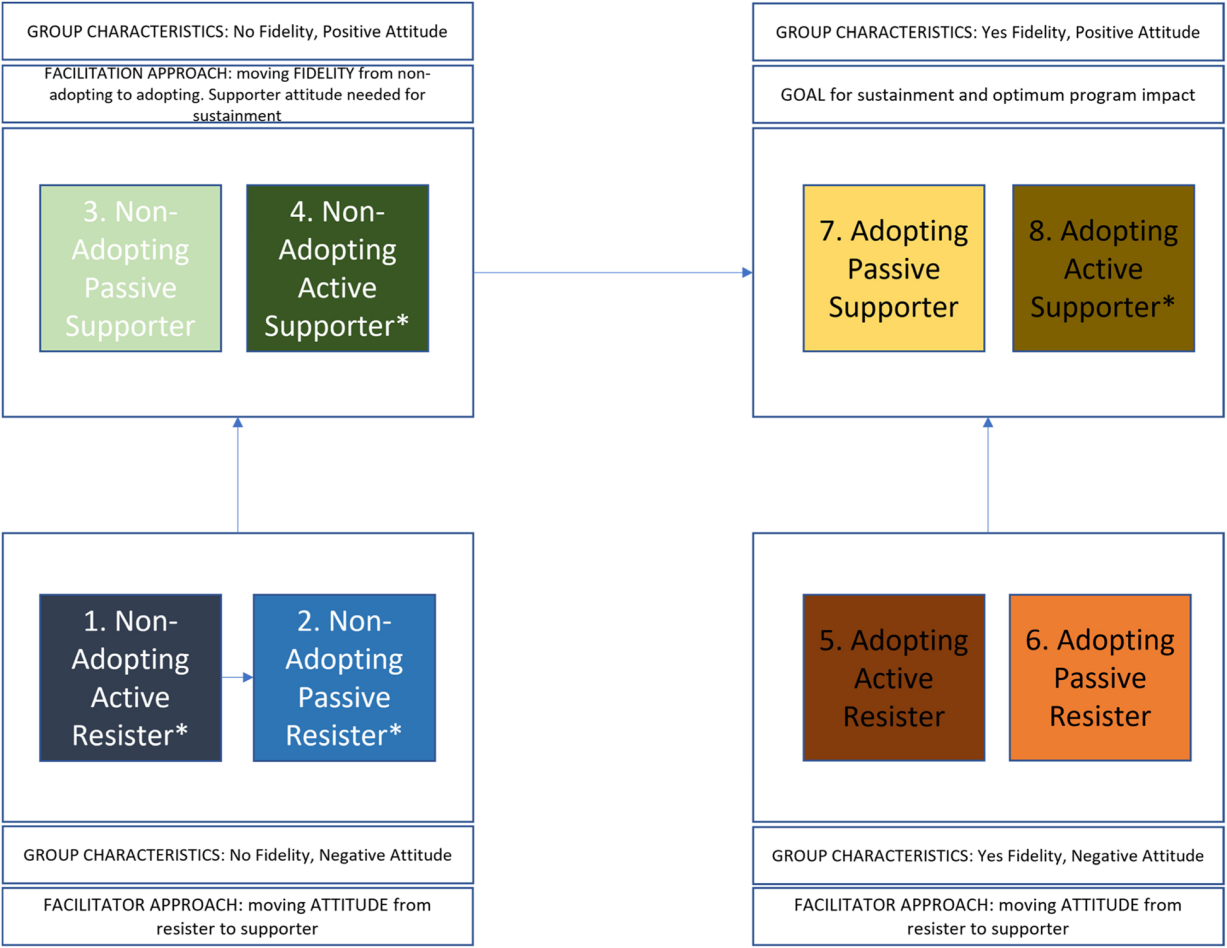
Facilitation approach by typology

Figure 3 presents how we believe the full classification system could inform tailoring of facilitation efforts in the future. This figure reflects strategies we used in our study and draws on motivational interviewing philosophies (e.g., assessing readiness, affirming values, exploring resistance), concepts from the trans-theoretical model of change (e.g., meeting people where they are), consultation with facilitation experts (e.g., attending VA facilitation office hours for case discussion), applying anecdotes from the VA facilitation training, brainstorming with the study team, and field experience. This figure illustrates that teachers without fidelity and a negative attitude (i.e., non-adopting resisters) are the prime target; the facilitation goal for this group is to support a shift *up* to a more positive attitude without attending to fidelity (at least initially). Similarly, the adopting resisters group has fidelity but a negative attitude. To prevent spreading a negative perception of the EBP in the context, this group is a secondary target for facilitation. As with the non-adopting resisters the goal is to attend to a shift *up* related to attitude, fidelity is not an issue for this group. When implementers have positive attitudes about the innovation but are not adopting with fidelity (i.e., non-adopting supporters), facilitation can focus on a shift *over* toward developing skills to reach fidelity, being careful to encourage and not undermine positive perceptions of the innovation. Our strategy reflects that influence may be a less malleable trait to target with facilitation; thus, it is important that the attitude of active resisters is addressed. Finally, adopting supporters can be connected with peers struggling to implement, acknowledged for their efforts, and encouraged to share their lessons learned with others.

## Discussion

The current study presents a classification approach for adopter behavior that considers adopter fidelity/adaptations, attitude, and influence. This approach goes beyond foundational categorizations based on the speed of adoption [30] to provide additional nuance for understanding implementers’ response to an implementation effort. Data from this study support the ability to apply the classification approach with moderate to high reliability and to use the approach to tailor facilitation toward improved fidelity of implementation. Further, qualitative data support distinct patterns of perceptions across the responder types as they relate to the innovation, implementation strategies, and context. Together, these findings suggest the potential of our approach for wider application and potential to improve clarity and standardization of the process for tailoring facilitation, a critical need for successful facilitation [31].

As illustrated by our field experience, not all response types included in the expanded classification approach may be present in a given study. In fact, some combinations are more logically plausible than others, depending on the context and innovation. For example, implementers with fidelity but a negative attitude and active influence may not be common (i.e., adopting active resisters). Yet, it is conceivable that medical and other professional contexts could have staff who comply with required policies while actively complaining about the inconvenience or burden of the EBP, thus undermining its effective implementation in the context. As another example, adopting passive resisters will appear compliant with EBP implementation but may be at risk for burnout. Awareness of the potential for all these combinations has advantages for the development and tailoring of implementation strategies for initial implementation and for the sustainment of EBPs. Specifically, we hypothesize that greater numbers of active supporters of an EBP (regardless of fidelity) would be important for sustainment. Without active supporters, an EBP is unlikely to endure in a context for the long term. Thus, our approach provides practical consideration to expand the goals of implementation strategies broadly and facilitation specifically beyond improving initial uptake and fidelity.

Implementation facilitation has been described as a “black box” [15] with over 72 distinct roles [32] and 22 “complex skills” [15]. Our approach provides practical common language and approaches for facilitators across contexts and innovations to help demystify the process. Specifically, this work led to the development of attitude cue lists to aid facilitators in classifying response, generation of adaptation examples that were fidelity-consistent and fidelity-inconsistent, and example scripts and responses for use with each response type. Facilitation operates along a continuum of task-focused to holistic enabling-individual focus [32]. Our approach suggests for whom which type of facilitation may work best. That is, resisters will likely be unresponsive to task-focused approaches and need enabling support such as a listening ear for reflection and connection to meaning of the implementation effort. In contrast, over-adapting adopters (i.e., non-adopting active supporters) have readiness and expectation to work with facilitators on their use of EBPs. Given how heavily the success of facilitation depends on the skills and strategies of the facilitator [15], our approach has potential utility to guide when and with whom certain skills are most needed.

In addition to providing a practical tool to guide facilitation, this work illustrates that there are nuances in fidelity not captured by quantitative scores alone. Specifically, not all low fidelity cases are the same, and different types of responses may be best served with different implementation strategies. Active resisters may benefit from strategies to enhance motivation whereas non-adopting supporters may need support for goal setting and/or making appropriate adaptations. Testing the effectiveness of approaches for tailoring implementation strategies is a promising area for future research [33]; our classifications could provide a clear foundation for such tailoring. Regardless, our data suggest that forming impressions about implementers’ behavior based on quantitative fidelity data alone can be misleading. Fidelity checklists may capture the “letter of the law” while failing to consider the “spirit.” Researchers and practitioners supporting frontline implementation efforts will likely benefit from gathering additional information to understand the factors underlying low fidelity scores. For example, other researchers have formed typologies of behavior specific to the Safewards innovation for mental health wards, which also considered whether adaptations were fidelity-consistent or fidelity-inconsistent [34]. The classification approach described in our work may provide a useful starting point for standardizing facilitation to the needs of implementers beyond our specific innovation.

Notable findings of our study include the relation between organizational context and response type as well as response type shifts and personal factors. Enthusiastic adopters were positive about their context, over-adapting adopters were neutral, and both types of non-adopters shared negative opinions and concerns about the context. Specifically, enthusiastic adopters shared that leaders were supportive, teachers discussed and supported one another in exploring ideas for WISE (e.g., hallway conversations and incorporated into staff meetings), and their site identified and implemented processes for institutionalizing WISE within their curriculum (e.g., added to lesson plans). Conversely, non-adopters shared challenges of staffing, receiving needed supplies, negative cultures around new programs, and lack of support from their site leadership. In i-PARIHS terms [26], these findings illustrate that factors of the context and recipients are difficult to address, even with robust innovations and facilitation. In some cases, individual and/or contextual capacity building may need to precede and/or be used conjointly with other implementation strategies. For example, Donmlyn et al. [35] propose a pre-implementation process of engagement, assessment, feedback and prioritization, and strategizing to increase readiness and capacity in general as well as specific to a particular innovation. Contextual barriers have been identified as critical across a range of settings and innovations [36–38]; ECE faces a number of contextual barriers of its own for use of EBPs, especially related to obesity prevention [39]. High rates of non-response using our classification approach may provide an indication of unaddressed contextual barriers.

The study includes limitations and strengths. One key limitation is the focus on the use of this approach with only one innovation and in limited contexts to date. The approach will have to be applied across other innovations and contexts to better assess its utility and reliability for improving implementation under different circumstances. Inter-rater reliability was highest in our study among the facilitators who had undergone the external VA training in implementation facilitation; acceptable but less robust consistency was demonstrated with the facilitator who received the adapted training indirectly. In addition, our interviews occurred in the fall and winter of the school year; an additional interview with a subset of teachers at the end of the school year could have provided evidence before or against shifts in response type that the researcher team perceived. Another limitation to the proposed approach is the reliance on the facilitator’s discretion to assess implementers’ attitudes. Facilitation, in general, relies on the communication, interpersonal, and assessment skills of the individual acting as the facilitator and can vary a great deal from facilitator to facilitator [27]. Finally, we caution that the labels for response types are for illustrative purposes to improve the provision of support, not to form negative conceptions of implementers. There are a number of valid reasons why an implementer may resist adoption of an innovation, and these labels are not designed to be value statements or undermine the experience of practitioners. The proposed approach could provide one way to bring common thinking and language to facilitators. In addition, fidelity measures could be paired with validated measures of implementation attitudes [27] and opinion leadership [29] to reduce subjectivity in classifying response types. In fact, measures of attitude and influence could be assessed in pre-implementation phases to provide guidance to facilitators as they begin implementation support. Strengths of our study include the assessment of inter-rater reliability, evaluation of change across time in response to the application of the approach, extensive training of facilitators, and qualitative data to promote depth of understanding for the classification types. Further, facilitators in our study had ongoing contact with educators for the entire school year, which allowed for the detection of subtlety in interactions.

## Conclusions

This work used quantitative and qualitative data to provide a nuanced approach to classify implementer behavior that goes beyond fidelity checklists alone. Our team aligned each category with specific facilitation strategies, which contributed to improved fidelity in our study. We then expanded upon this field experience to propose a classification approach that may have utility beyond our study. Specifically, this approach highlights that the combination of implementer attitude and influence may be more influential than fidelity alone, especially as it relates to the potential for sustainability of an innovation. Our work also underscores the importance of context for implementation. Specifically, non-response to implementation efforts commonly overlapped with contextual barriers. This suggests the potential for capacity building prior to and/or alongside implementation efforts in the early care and education setting.

## Data Availability

The datasets used and/or analyzed during the current study are available from the corresponding author on reasonable request.

## Abbreviations

ECE: Early care and education
EBP: Evidence-based practices
F/V: Fruits and/or vegetables
IS: Implementation science
i-PARIHS: Integrated Promoting Action on Research Implementation in Health Services
RAPICE: Rapid Assessment Procedure Informed Clinical Ethnography
WISE: Together, We Inspire Smart Eating
DOI: Diffusion of innovations
